# Gene expression profile in experimental frozen-thawed ovarian grafts treated with scaffold-base delivery of adipose tissue-derived stem cells

**DOI:** 10.1016/j.clinsp.2022.100066

**Published:** 2022-06-28

**Authors:** Luciana Lamarão Damous, Marcos Eiji Shiroma, Ana Elisa Teófilo Saturi de Carvalho, José Maria Soares-Jr, José Eduardo Krieger, Edmund C. Baracat

**Affiliations:** a Laboratório de Ginecologia Estrutural e Molecular (LIM-58), Departamento de Obstetrícia e Ginecologia, Hospital das Clínicas da Faculdade de Medicina da Universidade de São Paulo, São Paulo, SP, Brazil; b Laboratório de Genética e Cardiologia Molecular, Instituto do Coração (Incor), Faculdade de Medicina da Universidade de São Paulo, São Paulo, SP, Brazil

**Keywords:** Ovarian tissue transplantation, Gene expression, Fertility preservation, Stem cells, Rat, Qpcr, Quantitative Polymerase Chain Reaction, RT-PCR, Real-time Polymerase Chain Reaction, HE, Hematoxylin-Eosin, Actb, Actin, beta, B2m, Beta-2 microglobulin, Hprt1, Hypoxanthine phosphoribosyltransferase 1, Ldha, Lactate dehydrogenase A, Rplp1, Ribosomal protein, large, P1, VEGF, Vascular Endothelial Grown Factor, POI, Premature ovarian insufficiency, GC, Control Group, G1, Group 1, G2, Group 2, PBS, Phosphate-buffered saline, rASCs, Rat adipose- derived stem cells, DMEM, Dulbecco's modified Eagle's medium, DMSO, dimethyl sulfoxide, SD, Standard deviation of mean, RNA, Ribonucleic acid, Serpinb5, Serpin peptidase inhibitor, clade B (ovalbumin), member 5, Cxcl, Chemokine (C-X-C motif) ligand, Casp14, Caspase 14, LTa, Lymphotoxin alpha (TNF superfamily member 1), TNF-β, Tumor necrosis factor beta, ANG, Angiogenin, MSC, Mesenchymal stem cells, IL, Interleukin, MMP, Matrix metalloproteinase

## Abstract

**Purpose:**

Gelfoam scaffold is a feasible and safe non-invasive technique for Adipose tissue-derived Stem Cell (ASC)-delivery in the treatment of frozen-thawed ovarian autografts. This study seeks to analyze the genes expression profile of rat frozen-thawed ovarian autografts treated with scaffold-based delivery of adipose tissue-derived stem cells.

**Methods:**

Eighteen adult Wistar rats were distributed into three groups: Control (frozen-thawed only); Group 1 (G1) and Group 2 (G2) (frozen-thawed ovaries treated with culture medium or ASC, respectively). Both treatments were performed immediately after autologous retroperitoneal transplant with scaffold-based delivery. The ovarian grafts were retrieved 30 days after transplantation. Quantitative gene expression (qPCR) for apoptosis, angiogenesis, and inflammatory cytokines (84 genes in each pathway) were evaluated by RT-PCR. Graft morphology (HE), apoptosis (cleaved-caspase-3), neoangiogenesis (VEGF), and cellular proliferation (Ki-67) were assessed.

**Results:**

In grafts treated with ASC, the apoptosis pathway showed the highest number of genes over-regulated – 49 genes – compared to inflammation cytokines and angiogenesis pathway – 36 and 23 genes respectively, compared to grafts treated with culture medium. Serpinb5 family was highlighted in the angiogenesis pathway and Cxcl6 in the inflammation cytokines pathway. In the apoptosis pathway, the most over-regulated gene was Capsase14. ASC treatment promoted the reduction of cleaved caspase-3 in the theca internal layer and increased cell proliferation by Ki-67 in the granulosa layer without altering VEGF. A mild inflammatory infiltrate was observed in both groups.

**Conclusion:**

ASC therapy in rat frozen-thawed ovarian autografts promoted an abundance of genes involved with apoptosis and inflammatory cytokines without compromising the ovary graft morphology and viability for short time. Further studies are necessary to evaluate the repercussion of apoptosis and inflammation on the graft in the long term.

## Highlights

• The scaffold-based delivery therapy with adipose tissue-derived stem cells in the rat ovarian autografts seems to be the best option when compared to direct injection or systemic route.
• Ovarian grafts treated with adipose tissue-derived stem cells showed the highest number of genes over-regulated in the apoptosis pathway, compared to inflammation cytokines and angiogenesis pathway.
• Capsase14 was the most over-regulated gene in the apoptosis pathway.
• The treatment with adipose tissue-derived stem cells in ovarian grafts treated didn't compromise the ovary graft morphology and viability for short time.

## Background

Ovarian tissue transplantation is the most recent technique of fertility preservation and could be a successful option. This technique has gained space among the others, especially in children who will undergo gonadotoxic therapy, and has the advantage of not delaying the start of treatment or because a partner is not necessary for its execution [1,2]. Although there are currently several cases of gestations spontaneous worldwide after transplantation of ovarian tissue, whether spontaneous or not, its actual efficacy still needs to be elucidated because functional return rates are low and last for a short period of time. Randomized clinical trials are required [3,4].

In this current scenario, several preclinical studies have tested different types of treatments that optimize the revascularization of the graft in order to minimize the ischemic/reperfusion injury. The present group has been testing the use of cell therapy with stem cells derived from adipose tissue, a promising option in regenerative medicine for the recovery of different types of damaged tissues. In the field of cardiovascular and cerebrovascular disease, the regenerative potential use of Adipose tissue-derived Stem Cells (ASC) has been increasing interest for create a clonogenic/angiogenic microenvironment. However, more preclinical studies are required since the assessment of safety should be considered for its use in clinical trials in order to get rational conclusions about its clinical applications [5].

The transplantation of ASC on soluble collagen scaffolds has a better therapeutic effect than transplantation of ASC alone, to treat Premature Ovarian Insufficiency (POI) in a rat model [6]. For the ovarian graft, the scaffold-based delivery therapy of these cells seems to be the best option when compared to the direct injection or systemic route. Several experimental models have tested the use of scaffold-based therapy for the ovary with the most varied types of materials, such as hydrogel, Matrigel, soluble collagen, fibrin and Gelfoam 6, 7, 8, 9, 10, 11.

Gelfoam has proved to be rapidly revascularized both in vitro and in vitro with skin flaps and in vivo models with transgenic mice [12,13]. Previously, the present group tested different techniques of ASC-based therapy or cell-free therapy in the frozen-thawed ovarian grafts [14,15]. The authors noticed that the Gelfoam scaffold could be a feasible and safe non-invasive technique for ASC delivery in the treatment of frozen-thawed ovarian autografts. There were no changes in graft morphology and didn't promote enhancement in fibrosis and apoptosis [7].

Thus, the present study intends to continue the research line, after the standardization of the in vivo model of frozen-thawed graft treatment with ASC in a scaffold-based delivery therapy. This study seeks to analyze the genes expression profile of rat frozen-thawed ovarian autografts treated with scaffold-based delivery of adipose tissue-derived stem cells.

## Material and methods

The study was carried out at the Laboratory of Structural and Molecular Gynecology (LIM-58), Gynecology Discipline, Department of Obstetrics and Gynecology, Faculdade de Medicina da Universidade de Sao Paulo (FMUSP), with the cooperation of the Laboratory of Genetics and Molecular Cardiology/Heart Institute/FMUSP. The experimental procedures followed institutional guidelines for the care and use of laboratory animals and were approved by the Ethics Committee/FMUSP (protocol 190/10).

### Experimental design

The study sample consisted of 18 twelve-week-old adult female Wistar (Rattus norvegicus Albinus) rats. The animals had access to a breed-specific food formula and water ad libitum throughout the experiment and were kept under adequate sanitary, lighting (12/12h), and temperature conditions in the animal laboratory.

The animals were distributed in three groups with six animals each, as followed: 1) Control Group (GC): ovaries submitted to frozen-thawed process; 2) Group 1 (G1): frozen-thawed ovaries autografts treated with culture medium, and 3) Group 2 (G2): frozen-thawed ovaries autografts treated with ASC.

### Cell isolation, ex vivo expansion, and ASC characterization

Inguinal subcutaneous adipose tissue was collected under sterile conditions from one 10-week-old male Wistar rat and rinsed with Phosphate-Buffered Saline (PBS). Rat Adipose-derived Stem Cells (rASCs) were isolated, characterized, and maintained in culture as previously described [16,17].

The harvested tissue was dissociated by digestion with 0.075% type IA collagenase (Sigma-Aldrich, Inc.) for 45 min. Enzyme activity was stopped, and the cell suspension was centrifuged at 300g for 15 min. Pelleted cells were recovered and plated onto 10-cm culture plates (NUNC, Rochester, NY). At 24h intervals, cultures were washed with PBS to remove contaminating erythrocytes and other unattached cells and then reefed with fresh medium. The plating and expansion medium consisted of low-glucose Dulbecco's Modified Eagle's Medium (DMEM) supplemented with 10% Fetal Bovine Serum (FBS) and penicillin/streptomycin antibiotics (Invitrogen Corporation, Carlsbad, CA).

Cells were maintained at 37°C with 5% CO_2_ in tissue culture dishes and fed twice a week until they reached 80% of confluence ‒ usually within 5 to 7 days after the initial plating. Once 80% confluence was reached (Passage 0), adherent cells were detached with 0.25% trypsin-EDTA (Vitrocell Embriolife, Campinas, SP, Brazil) and were either replated at 1 × 10^4^ cells/cm^2^ or used for experiments. Cultures were passaged every 3 to 5 days and used for experimental procedures until Passage 3.

The morphological and replicative characteristics as well as the immunophenotype (CD90+, CD29+, CD44+, CD73+, CD31-, CD45-) of the ASCs have been previously described in the present study's laboratory [18]. Although the characterization has been performed extensively in Mouse Adipose-derived Stem Cells (mASCs), the group conducted some immunophenotyping assays in rat Adipose-derived Stem Cells (rASCs). The percentage of CD90- and CD29-positive cells, the primary markers of a variety of various adult stem cells, was found to be approximately 90% (92.65% and 98.89%, respectively) in rASCs at the third culture passage (unpublished data from Nakamuta et al.).

### Vaginal smear collection

Before the experiment, vaginal smears were obtained daily. Only those animals showing at least two consecutive normal 4- to 5-day vaginal estrous cycles were included in the experiment. Two investigators blinded to the experimental treatments performed this analysis (LLD and MES). In case of doubt or discordant analysis, a third investigator (JMS) was requested.

The vaginal smear was obtained with a swab soaked in physiological solution and placed on a standard slide and immediately fixed in absolute alcohol for staining using the Shorr-Harris technique. The slides were analyzed under a light microscope at 10 × and 40 × magnification. Based on the proportion of cells found in the smears, the estrous cycle phases were characterized as follows: (1) Proestrus, the predominance of nucleated epithelial cells; (2) Estrus, the predominance of anucleated, keratinized cells; and (3) diestrus, the same proportion of leukocytes and nucleated, keratinized epithelial cells.

The ovarian transplant (G1 and G2) e oophorectomy (GC) was performed during the diestrous phase and euthanasia between day 30 and day 35 during always in diestrus [14,15,19].

### Collection of ovarian tissue (oophorectomy)

Wistar female rats were anesthetized intraperitoneally with xylazine and ketamine at a dose of 15 mg.kg^−1^ and 60 mg.kg^−1^ of body weight, respectively. After the opening of the abdominopelvic cavity, the ovaries were identified, and their pedicles were clamped and immediately ligated with 4‒0 nylon sutures. The fallopian tubes were resected with the periovarian adipose tissue fragments. The ovaries were placed into cryovials until the cryopreservation is performed. The wall closure was performed with a 5‒0 nylon monofilament thread on two planes, the peritoneum-aponeurotic muscle, and the skin.

### Ovarian cryopreservation and thawing

After bilateral oophorectomy, the fresh ovary was immediately frozen in a slow cooling freezer. The whole ovaries were placed in 1.2-mL cryovials (Sigma-Aldrich®, Inc.) with M2 medium with HEPES without penicillin and streptomycin (M2-Sigma-Aldrich®, Inc.) and dimethyl sulfoxide (DMSO) (Sigma-Aldrich®, Inc.) 1.4 M as cryo- protector and held at room temperature for 5-min. The cryovials were sealed by twisting their caps, placed in a temperature-programmed freezer (CL-8800, Cryogenesis software, Freezer Control) and cooled from 25° to 10°C at 1°C/min, then at a rate of 0.5°C/min to -7°C, and maintained at -7°C for 5-min. Ice nucleation was induced manually using pre-cooled forceps, and the temperature was held at -7°C for a further 5-min for the release of latent heat fusion. The tissue was frozen at -55°C at a rate of 0.5°C/min, plugged in liquid nitrogen at -196°C, and stored for 24h [20].

For thawing, the cryovials were removed from liquid nitrogen and held at room temperature until the ice melted. The ovaries were washed two times for 5-min in a fresh M2 medium, and gently shaken to remove cryoprotectant before further processing. The ovaries were maintained in M2 at room temperature until the transplant [20]. Six animals were used as a control for the frozen-thawed process (GC).

### Ovarian transplantation and ASC therapy with scaffold-base delivery

A second laparotomy was performed utilizing the same technique previously described. Each animal received a pair of autologous ovary transplants. With a simple stitch of 4‒0 nylon suture, intact whole ovaries were implanted in the retroperitoneum in the proximity aorta and vena cava, without vascular anastomosis, each on one side of the psoas muscle.

At this point, the animals were distributed according to the treatment, as follows (n = 6 for each group): Group 1 (G1): Gelfoam with 15 μL of DMEM solution/ovary, and Group 2 (G2): Gelfoam with ASCs at a concentration of 5 × 10^4^ cells in 15 μL of vehicle/ovary. The Gelfoam was applied to the ovary without a stitch. Both grafts from each animal received the same treatment. The number of cells per graft and the dose of 15 μL were standardized in previous studies of the present group [7,19] .After the treatments, the wall closure was performed using a 5–0 nylon monofilament thread on two planes, including the peritoneum-aponeurotic muscle and the skin.

### Graft retrieval

The animals underwent a third surgical procedure between 30 and 35 postoperative days, always in the diestrus phase. The abdominal cavity was opened, and the ovaries were macroscopically identified and assessed. The muscle bed was also assessed for vascularization and surrounding adhesions. The grafts were subsequently removed whole. Following this procedure, the animals were euthanized with a lethal dose of the previously used anesthetics. One side of the ovarian graft was immediately plugged-in liquid nitrogen at -196°C and stored until analysis. In groups G1 and G2, the other side of the graft was processed for immunohistochemical and hematoxylin-eosin.

### Quantitative PCR

Total RNA was extracted from thawed ovaries using the QIAzol Lysis Reagent and the RNeasy Micro Kit (Qiagen, Hilden, Germany) according to the manufacturer's instructions. The solution was treated with the RNase-Free DNase Set (Qiagen, Hilden, Germany). The total RNA obtained from each sample was quantified spectrophotometrically (ND100 NanoDrop®, Thermo Fisher Scientific Inc. Co.), and the RNA integrity was assessed by electrophoresis on a 1% agarose gel.

Total RNA (1 μg) purified from each sample was transcribed into cDNA via reverse transcription using the RT^2^ First Strand Kit (Qiagen, Hilden, Germany) according to the manufacturer's instructions. The synthesized cDNA underwent reaction in qPCR in PCR array plates specific for each pathway: angiogenesis (RT^2^ Profiler PCR Arrays, cat. PARN-024Z, Catalog#330231, QIAGEN-SABiosciences Corporation, USA), apoptosis (RT^2^ Profiler PCR Arrays, cat. PARN-012Z, Catalog#330231, QIAGEN-SABiosciences Corporation, USA) and inflammatory cytokines (RT^2^ Profiler PCR Arrays, cat. PARN-011Z, Catalog#330231, QIAGEN-SABiosciences Corporation, USA) and the 7500 Real-Time PCR System (Applied Biosystems, CA, USA) were used. The list of 84 gene profiles analyzed for each pathway is detailed in Additional file 1 (Genes Profile). The qPCR results were calculated using the ΔΔCT method and specific SABiosciences software. The gene expression results are provided as fold changes, relative to the reference group of intact ovaries (GC). The software assigns this group (intact ovaries) a value of 1, due to ΔΔCT relative expression analysis method [21]. So, no value of fold expression lower than 1 might be considered significant.

Genes with fold regulation > 2 were considered upregulated and those with values < 2 were considered downregulated. The values were obtained for statistical analyses, but relative expression reflects the number of times that a specific gene was expressed compared to a reference sample or group and a significant value was considered to be a threefold change relative to the fresh ovary. All gene expression levels were normalized using the average of the housekeeping genes (Actb, B2m, Hprt1, Ldha, and Rplp1) following the software manufacturer's instructions. Data were analyzed using Web Basis Data Analysis at https:// www.google.com/url?sa=t&rct=j&q=&esrc=s&source=web& cd=2&cad=rja&uact=8&ved=2ahUKEwi_mPepoKTeAhUF TZAKHa9LA2IQFjABegQICRAB&url=https%3A%2F%2F dataanalysis.sabiosciences.com%2Fpcr%2Farrayanalysis.php %3Fwuid%3D8fa7191b-bb49-409d-b896-8ce28966b04e%26 logindata%3D%26customerdata%3D%26customeremail%3D %26platform%3DcustomArray%26format%3DX&usg=AOv Vaw23-l6Q74gr7qsQXNr95B-L.

### Immunohistochemical assay

Sections containing ovarian stroma were immunostained to measure apoptosis via cleaved-caspase-3 expression (SANT-SC-1226, 1:100, Santa Cruz Biotechnology, Inc. Santa Cruz, CA, EUA), angiogenesis by VEGF (SANT-SC-152, 1:100, Santa Cruz Biotechnology) and cellular proliferation by Ki-67 (M724001-2, 1:100, Dako North America Inc., Carpinteria, CA, USA). For the negative controls, the primary antibody was omitted to avoid bias. Cross-sections embedded in paraffin were treated with antigenic exposure and blocked with 2% casein in PBS. The tissue sections were incubated with the respective primary antibodies overnight at 4°C. The sections were incubated with a biotinylated rabbit secondary antibody (universal polymer anti-mouse and rabbit Histofine® 1:400, Vector Laboratories, Burlingame, CA, USA) and streptavidin peroxidase followed by the peroxidase substrate diaminobenzidine tetrahydrochloride according to the manufacturer's instructions.

The red-brown coloring of the cytoplasm/nucleus was considered positive staining (any other coloring was considered negative staining). Negative controls were used to avoid bias. For the negative controls, the primary antibody was omitted in each different immunohistochemistry staining.

Images of the sections were obtained using an image acquisition software system (Leica DM2500), and measurements were made using Leica QWin V3 software. A red-brown coloring of the cytoplasm/nucleus was specified as positive staining (otherwise as negative staining). The assessment was performed by counting the number of total cells and the positive cell staining in granulosa cells and theca internal layer of antral follicles at × 200 magnification, and the results are expressed as a percentage of the positive area (arbitrary unity/mm^2^) [14,19]. Two independent investigators blinded to the experimental treatments performed all measurements.

### Morphological analyze

Morphological evaluation was achieved through descriptive analyses of the grafts. Assessment of follicular quality was based on cell density, the presence or absence of pyknotic bodies, and the integrity of the basement membrane and of the oocyte. According to these criteria, follicles were classified as normal or degenerated; only the former were characterized and quantified [14].

The viable follicles were classified as follows: (1) primordial follicle, exhibiting only an oocyte and a layer of squamous cells; (2) primary follicle, exhibiting an oocyte and a layer or more of cuboidal or prismatic cells but no antrum; and (3) secondary follicle, exhibiting an oocyte and an antrum. The mature follicle was that which contained an oocyte with a voluminous antrum. The corpus luteum was that which had intact luteal cells containing a voluminous nucleus and surrounded by capillary blood vessels.

The ischemic injury was assessed as standardized in previous studies through the morphological analysis of findings such as monomorphonuclear inflammatory infiltrate, neutrophilic exudate, ischemic alterations (tissue necrosis), and congestion (vascular obliteration) the inflammatory infiltrate was classified as mild, moderate, and severe [22].

For fibrosis evaluation, the slices were stained with Picrosirius Red, and measures were performed in eight different fields per animal in the ovarian stroma, at 400 × magnification, and the results are expressed as a percentage of the positive area (arbitrary unity/mm^2^).

### Statistical analysis

According to the Shapiro-Wilk normality test for normal distribution, an unpaired *t*-test was utilized to compare immunohistochemical analysis between different treatment groups (vehicle or ASC). The results were expressed as mean ± Standard Deviation of the mean (SD). All statistical analyses were performed using Graphpad Prism 7.0 (Graphpad Software Inc., CA, USA). p values lower than 0.05 were considered significant.

## Results

**Figure 1:**
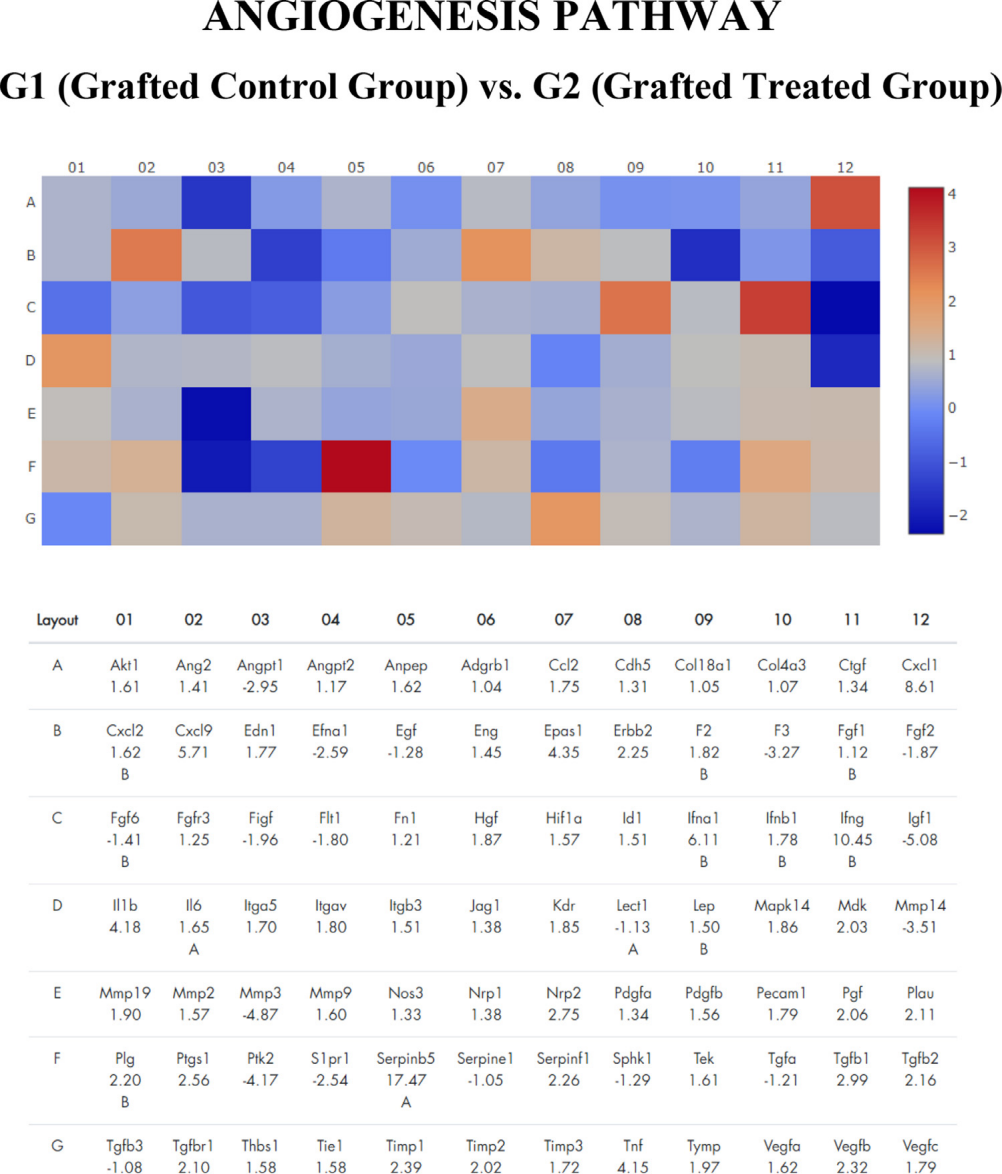
Gene expression profile of angiogenesis pathway of frozen-thawed rat ovarian autografts treated with Adipose tissue-derived Stem Cells (ASC) (Group 2) compared with autografts treated with culture medium (Group 1). Genes with fold regulation > 2 were considered upregulated and those with values < 2 were considered downregulated. In red, genes upregulated. In blue, genes down regulated.

**Figure 2:**
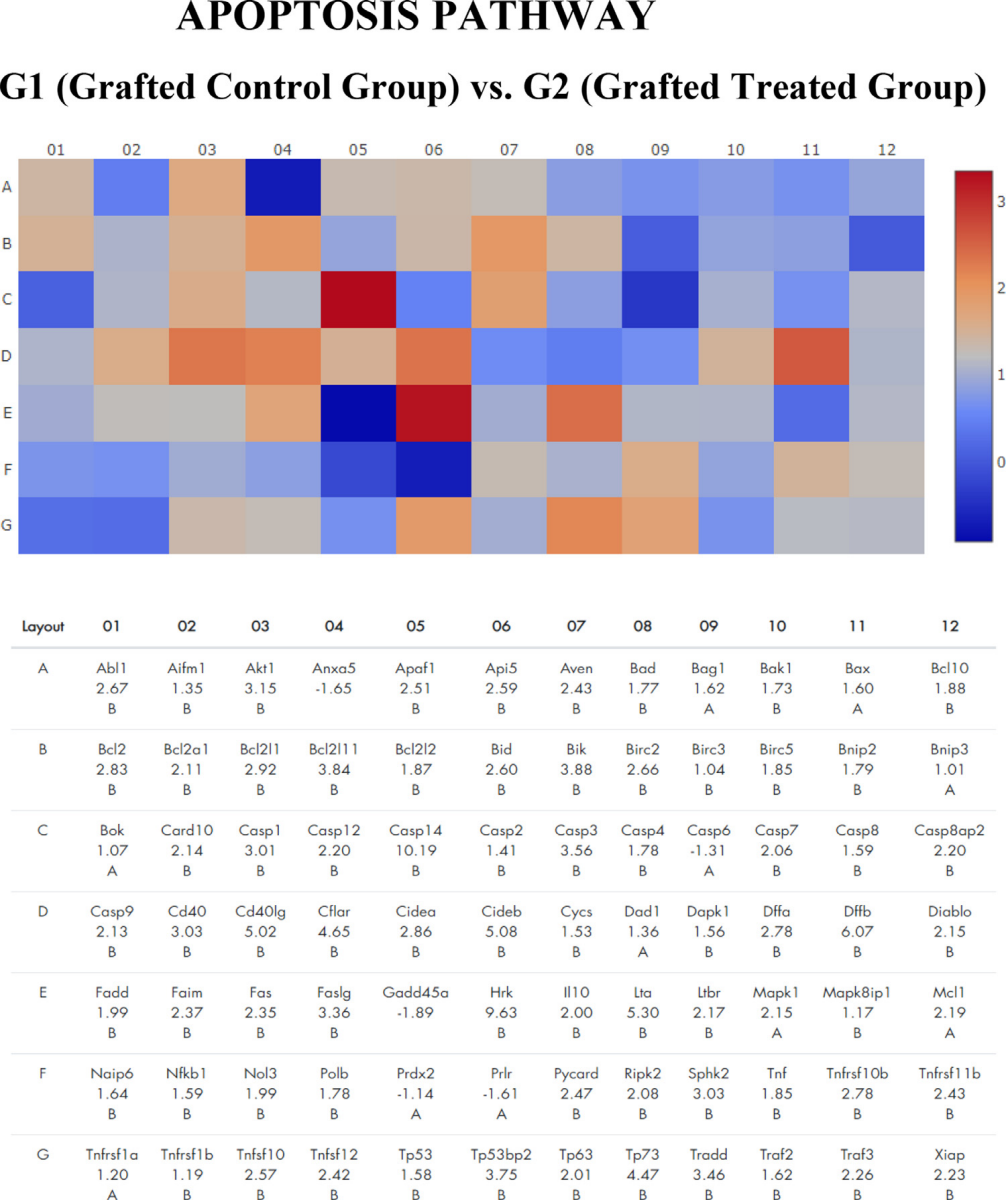
Gene expression profile of apoptosis pathway of frozen-thawed rat ovarian autografts treated with Adipose tissue-derived Stem Cells (ASC) (Group 2) compared with autografts treated with culture medium (Group 1). Genes with fold regulation > 2 were considered upregulated and those with values < 2 were considered downregulated. In red, genes upregulated. In red, genes upregulated. In blue, genes down regulated.

**Figure 3:**
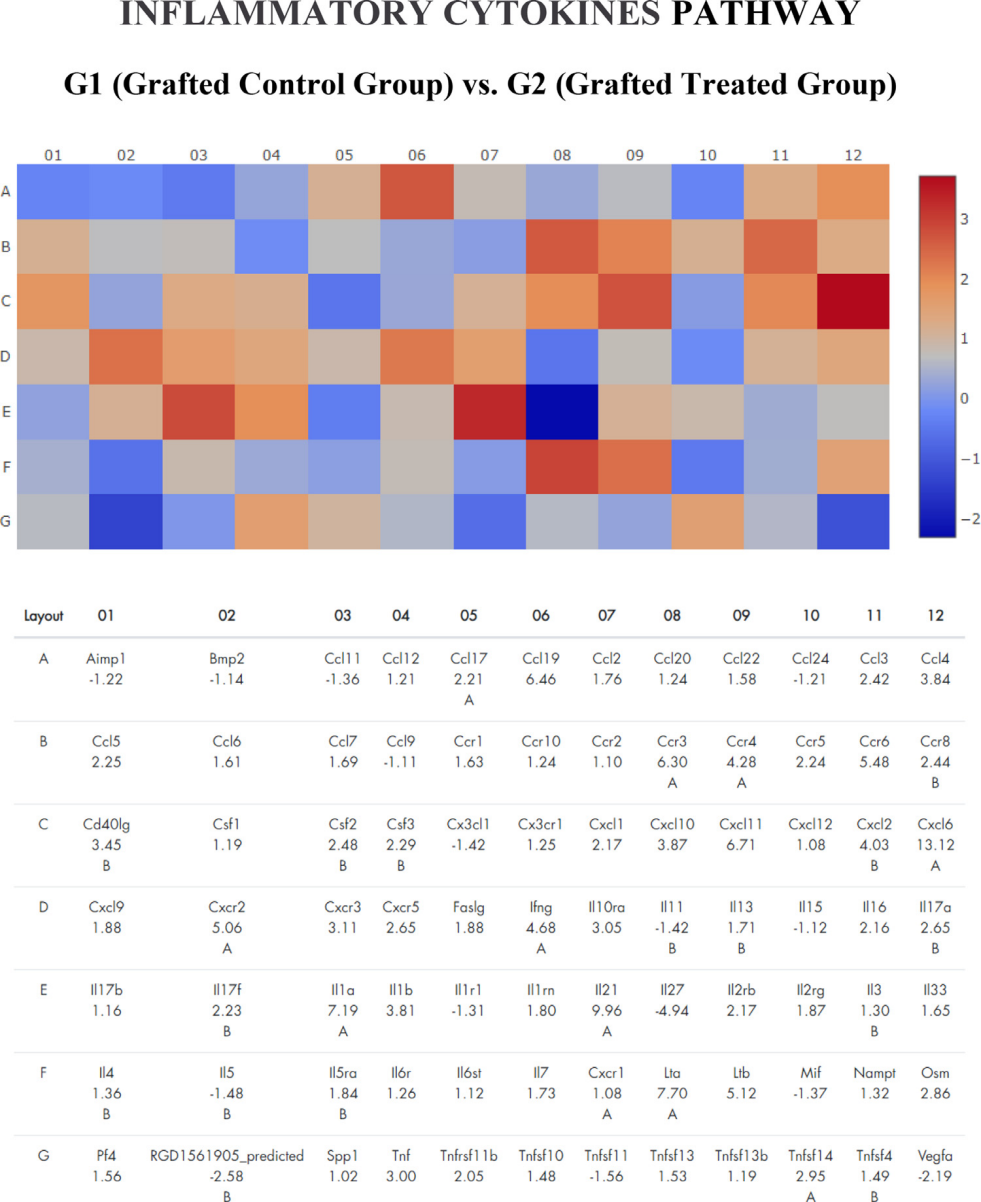
Gene expression profile of inflammatory cytokines pathway of frozen-thawed rat ovarian autografts treated with Adipose tissue-derived Stem Cells (ASC) (Group 2) compared with autografts treated with culture medium (Group 1). Genes with fold regulation > 2 were considered upregulated and those with values < 2 were considered downregulated. In red, genes upregulated. In blue, genes down regulated.

The number of over-and under-regulated genes in each pathway is represented in Figures 1, 2 and 3. Table 1 shows the tops ten over-regulated with their respective Fold Changes. The apoptosis pathway was the one that showed the highest number of over-regulated genes – 49 genes – compared to inflammation cytokines and angiogenesis pathway – 36 and 23 genes, respectively.

**Table 1:** Top ten most up- and down-regulated genes in frozen-thawed rat autografts treated Adipose tissue-derived Stem Cells (ASC) (G2) compared with autografts treated with culture medium (G1).

	Angiogenesis		Apoptosis		Inflammatory cytokines	
	23 Over	8 Under	49 Over	0 Under	36 Over	3 Under
	Gene	FR	Gene	FR	Gene	FR
**Top ten Over-Expressed genes**	Serpinb5	17.47	Casp14	10.19	Cxcl6	13.12
	lfng	10.45	Hrk	9.63	Il21	9.96
	Cxcl1	8.61	Dffb	6.07	Lta	7.70
	lfna1	6.11	Cideb	5.08	Cxcl11	6.71
	Cxcl9	5.71	Cd40lg	5.02	Ccl19	6.46
	Epas1	4.35	Lta	5.30	Ccr3	6.30
	Il1b	4.18	Cflar	4.65	Ccr6	5.48
	Tnf	4.15	Tp73	4.47	Ltb	5.12
	B2m	3.12	Bik	3.88	Cxcr2	5.06
	Tgfb1	2.99	Tp53bp2	3.75	lfng	4.68
**Top ten Under-Expressed genes**	Igf1	5.08			Il27	4.94
	Mmp3	4.87			RDG	2.58
	Ptk2	4.17			Vegfa	2.19
	Mmp14	3.51				
	F3	3.27				
	Angpt1	2.95				
	Efna1	2.59				
	S1pr1	2.54				

Serpinb5 family was highlighted in the angiogenesis pathway (Fold Regulation 17.47) and Cxcl6 in the inflammation cytokines pathway (Fold Regulation 13.12). In the apoptosis pathway, the most over-expressed gene was Capsase14 (Fold Regulation 10.19). By analyzing the 10 most over-regulated genes in each pathway, it is observed that Cxcl family genes are common in the angiogenesis (Cxcl1 and Cxcl9) and inflammatory cytokines (Cxcl 6 and Cxcl11) pathway.

**Figure 4:**
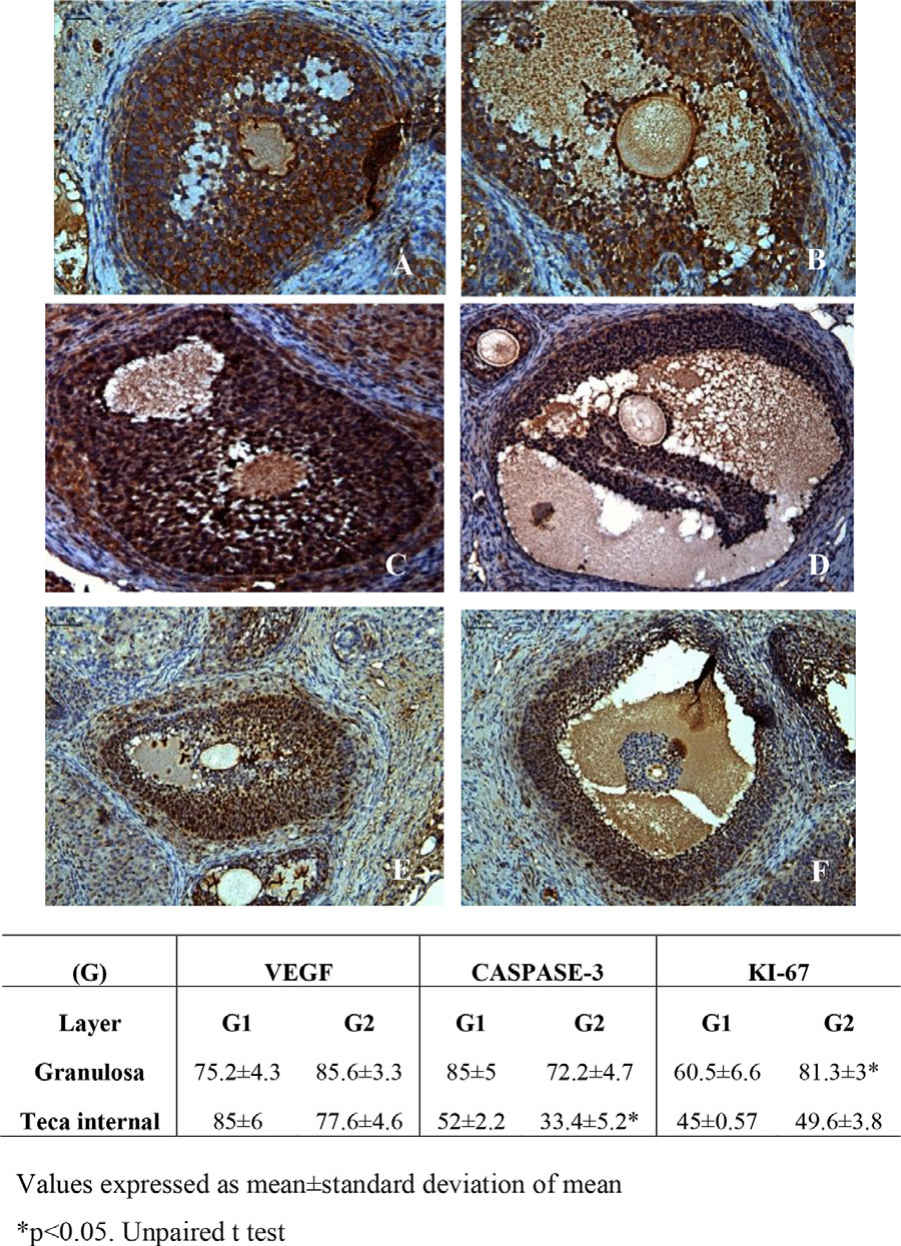
Photomicrographs of frozen-thawed rat ovarian autografts by immunohistochemistry for VEGF (A and B), apoptosis by Cleaved Caspase-3 (C and D) and cellular proliferation by Ki-67 (E and F) treated with culture medium (A, C and E) or adipose tissue-derived stem cells (B, D and F) (200 ×). (G) Immunohistochemical analyzes for angiogenesis (VEGF), apoptosis (Cleaved caspase-3) and cellular proliferation (Ki-67) in frozen-thawed rat autografts treated with culture medium (G1) or adipose tissue-derived stem cells (G2). The assessment was performed by counting the number of total cells and the positive cell staining in granulosa cells and theca internal layer of antral follicles. Values are expressed in percentage of positive area (arbitrary unity/mm^2^). *p < 0.05, Unpaired *t*-test.

**Figure 5:**
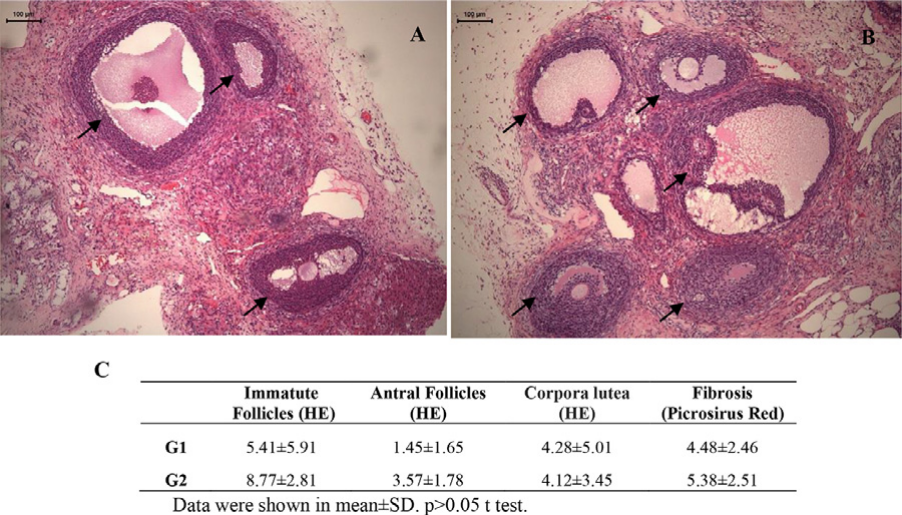
Photomicrographs of frozen-thawed rat ovarian autografts treated with culture medium (A) or adipose tissue-derived stem cells (B). In (C) follicular density and fibrosis quantification. Arrows shows viable ovarian follicles (HE 100 ×).

ASC treatment promoted the reduction of cleaved-caspase-3 in the theca internal layer and increased cell proliferation by Ki-67 in the granulosa layer without altering VEGF (Fig. 4). The morphology of ovarian fixed and stained material by HE was similar between groups. Ovarian follicles were observed in several maturation stages (primordial, primary, preantral and antral), as well as whole corpora lutea, but some degenerated. Ovarian follicles density was similar between groups and a mild inflammatory infiltrate was observed in both groups (Fig. 5).

## Discussion

Ovarian transplantation is one of the most recent in comparison to other organs with established methodology. In the avascular transplantation of the ovary, once the initial hypoxia is overcome until the creation of a new vascular network, there is massive activation of primordial follicles in this graft with the consequent premature exhaustion and short survival, which is the main limiting factor of ovarian transplantation [23,24]. Several strategies have been proposed with the aim of maintaining graft homeostasis in the initial phases after functional return, with a balance between apoptosis and proliferation, in addition to the self-limited action of inflammatory markers until neoangiogenesis. In this context, the genetic profile of the cryopreserved ovarian graft treated with ASC shows several mechanisms involved in the mechanism of adaptation of the ovarian graft as apoptosis, angiogenesis, and inflammatory cytokines. However, there were some genes that had a greater abundance of RNA than groups not treated with ASC. Serpinb5 gene was prominent in the angiogenic pathway. Protease inhibition by serpins controls an array of biological processes, including inflammation. Serpinb5 is a tumor suppressor that binds directly to extracellular matrix components, suggesting that the surface binding interaction is responsible for the inhibition of tumor-induced angiogenesis. Clinically, this gene may play an important role in rectal cancer progression and response to neoadjuvant and serve as a novel prognostic factor [25]. The Serpinb5 RNA abundance may protect the graft against tumor risk of ASC through cellular suppression of excess of neoangiogenesis, which may reflect on the VEGF expression and ovarian morphology.

Several Cxcl family genes are chemotactic cytokines, and also appear among the main over-expressed genes: Cxcl1 and 9 in the angiogenesis pathway and Cxcl6 and 11 in the inflammation pathway. With proinflammatory action on the immune system and also homeostatic and neoangiogenesis, this family of genes can have fundamental action on the mechanism of adaptation to the graft. Cxcl1 and 9 are cytokines with primarily proinflammatory action, but curiously they stand out in the angiogenesis and noninflammatory pathways [26,27], which shows that these pathways are closely related in the process of adaptation of the ovarian graft, in which it seems to trigger first an apoptotic response, with subsequent activation of the inflammatory cascade and recruitment of specific leukocytes for humoral defense and with subsequent induction of angiogenesis for the survival of this avascular graft. Although the ovarian transplant is autologous, it is heterotopic and avascular, and it is necessary to create a vascular network around the graft that is established within 48 hours after transplantation [1, 2, 3, 4,23]. This graft adaptation of the host can explain this initial increase of the genes involved in the inflammatory cascade that later gives space for cellular proliferation and neoangiogenesis. This hypothesis is reinforced by increased cell proliferation in the granulosa layer in ASC-treated grafts.

Casp14 was the most overexpressed gene in the apoptosis pathway. The real role of caspase-14 remains unclear in ovaries. Evidence sheds light on the crucial role of caspase-14 in the skin, with highly differentiated cornified areas of lung squamous cell carcinoma and cervix carcinoma. It is now clear that the casp-14 – activating protease is not a caspase but probably an epidermis-specific serine protease with elastase-like properties [28]. In vitro study evaluating the expression and function of casp-14 in lung, adenocarcinomas suggest that casp-14 is an anti-apoptotic protein targeting apoptosis-inducing factors [29]. This function of Casp-14 in other tissues makes us assume that ovarian transplantation it would improve graft adaptation. Reinforcing this data, the quantification of collagen fibers showed no difference between grafts treated with ASC.

The LTa gene appears over-expressed both in the apoptosis and inflammatory pathway. This gene produces TNF-β which is involved in the regulation of cell survival, proliferation, differentiation, and apoptosis [30]. It shows that all pathways studied are closely related.

In another experiment to identify the key secreted factors in ovarian grafts co-transplanted with bone marrow-derived mesenchymal stem cells, Angiogenin (ANG) was one of the most robustly up-regulated proteins [10]. This finding differs from ours, in which ANG does not appear among the top 10 molecules over-expressed. A limiting factor in this study is that the ovarian tissue for xenotransplantation was obtained from a single patient who underwent gender reassignment surgery and the Human MSCs were isolated from the bone marrow of a healthy female. These methods are different from ours, an autotransplantation of ovarian rats and the ASC were obtained from adipose tissue

There are currently seven known CXC chemokine receptors in mammals, named CXCR1 through CXCR7 [26,27,31]. In the present study, CXCR2 was higher only in inflammatory pathway and this difference in subtype result may be due to the earlier evaluation performed by these authors – 7 days after transplantation ‒ while the model was later, after 30 days. These dates were standardized in previous studies [14,19]. Another difference is the use of the Gelfoam scaffold used in the present model. However, previous studies from the present group have shown that a scaffold based on Gelfoam's sponge does not increase inflammation and fibrosis in ovarian grafts [7]. The morphological analysis of the grafts showed that the inflammatory infiltrate is similar in both groups, treated or not with ASC.

The Interleukin 1 beta highlighted in the angiogenesis pathway was previously demonstrated in high levels acutely in rats with focal ischemia [32]. Another Interleukin family gene highlighted was Interleukin 21 in the inflammatory pathway. There are two general areas where the positive effects of IL-21 are important: cancer immunotherapy and the development of memory responses to various viral pathogens. Conversely, IL-21/IL-21R blockade may also have therapeutic benefits in the treatment of autoimmune diseases and inflammatory conditions [33]. A recent study suggests that targeting the IL-21/IL-21R signaling axis may provide a novel approach for the development of new therapeutic agents for the prevention of parasite-induced immunopathology and tissue destruction [34]. Therefore, the increase in IL-21 induced by ASC can compromise the viability of the ovarian graft and new treatment strategies can be developed from the study of canonical routes and their suppression.

In the angiogenesis pathway, two genes of Matrix Metalloproteinase (MMP) were under-regulated – MMP3 and MMP14. Proteins of the MMP family are involved in the breakdown of extracellular matrix in normal physiological processes, such as embryonic development, reproduction, and tissue remodeling, as well as in disease processes, such as arthritis and metastasis. Deficits in MMP14 lead to premature aging, short lifespan, and cell senescence in mice, suggesting an important role of MMP14 in extracellular matrix remodeling during aging. Most MMPs are secreted as inactive pro-proteins which are activated when cleaved by extracellular proteinases [35]. The gene expression profile of the healing tissue around nanotextured implants differed from that around machined-surface implants or control empty holes. Six MMP family genes were changed, including Mmp3 [36]. similar to the present results. Thus, the under-regulation of these family genes noted in the present study could be a protective effect against fibrosis formation, which could compromise the functionality of the ovarian graft. Therefore, a long-term study is necessary to see the impact of this result.

The increase in another family gene of MMP was also described in an experimental model of human ovarian tissue xenotransplants in nude mice – the MMP9, in addition to inflammatory cytokines of the family of Interleukins and CXCR, especially IL8 and CXCR4 [37]. This MMP9 increase appears to be beneficial to the ovarian graft by inducing a self-limited but essential neoangiogenesis within the first few hours after implantation, in which the avascular graft is vulnerable to hypoxia.

In a study of co-culture with ASC and different human breast cancer cell lines (BRCAs), the ASC significantly affect multiple gene expression like genes of CxCl, MMP and Serpine, similar to the present findings. However, these in vitro results can only give a hint towards the more complex situation in vivo and should be reproducible and validated in an in vivo model such as ours [38].

Kidney and liver transplants are among the most studied and may provide clues about the mechanisms involved in the transplantation of other organs, including those of the ovary. Various markers have been studied in an attempt to identify a specific indicator of graft rejection and graft acceptance after liver transplantation. Considering acute rejection, the most studied markers are pro-inflammatory and immunoregulatory cytokines and other proteins related to inflammation. Despite most of them showing an increased expression during acute cellular rejection, many of these cytokines cannot differentiate between rejection and infections, making their utility limited in clinical practice [39].

A limitation of the present study is that the analysis of the gene expression was performed in the total RNA of the ovarian graft and not in the follicular cells, i.e., the abundance of messenger RNA can be of specific components such as internal teak cells, granulosa cells, interstitial cells, fibroblasts, and other components of the ovarian matrix. In addition, other routes may not be identified by the same fact, which limits the interpretation of the data. In addition, the present study evaluates the acute process of the ovarian graft adaptation, immediately after the initial critical period of hypoxia until its revascularization. Once the mechanism of this acute phase has been elucidated, long-term studies can evaluate its functionality, both from an endocrine and reproductive point of view. The present study showed that there is an abundance of RNA involved in graft adaptation and in the control of neoangiogenesis, apoptosis, and inflammatory process, which suggest better graft adaptation when treatment with scaffold-base delivery of tissue-derived adipose stem cells, at least short term.

In an experimental model to evaluate rejection after liver and cardiac transplantation, the expression of the inflammatory set of genes returned to the baseline at a later time after transplantation. These authors suggest monitoring the graft expression at different times in order to identify the gene set differentiation between rejection and tolerance [40]. In the same way as in other organs, in ovarian tissue transplantation, a cascade of intricate events appears to occur, so that a single biomarker cannot be able to reflect all the alterations associated with ovarian graft. Therefore, a panel of different biomarkers will be needed to properly evaluate the inflammatory suppression and anticipate the trigger of neoangiogenesis, and also to make this a self-limited process once the new vascular network is established.

## Conclusion

ASC therapy in rat frozen-thawed ovarian autografts promoted an abundance of genes involved with apoptosis and inflammatory cytokines without compromising the ovary graft morphology and viability for short time. Further studies are necessary to evaluate the repercussion of apoptosis and inflammation on the graft in the long term.

## Ethics approval and consent to participate

Not applicable.

## Availability of data and materials

All data generated or analyzed during this study are included in this published article.

## Authors' contributions

LDD: Conceptualization, Data curation, Formal analysis, Methodology, Investigation, Writing original draft. AETSC and MES: Datacuration, Investigation and Methodology; JMS: Project administration, Supervision and Writing - review & editing. JEK: Supervision and Validation. ECB: Conceptualization, Funding acquisition and Validation.

## Funding

The study was supported by São Paulo Research Foundation (FAPESP) for grant support (Process numbers: 2010/17897-5 and 2012/09469-9).

## Conflicts of interest

The authors declare no conflicts of interest.
